# Study of the Microstructure Evolution of Low-pH Cements Based on Ordinary Portland Cement (OPC) by Mid- and Near-Infrared Spectroscopy, and Their Influence on Corrosion of Steel Reinforcement

**DOI:** 10.3390/ma6062508

**Published:** 2013-06-18

**Authors:** José Luis García Calvo, Mercedes Sánchez Moreno, María Cruz Alonso Alonso, Ana Hidalgo López, Juan García Olmo

**Affiliations:** 1 Institute for Construction Sciences Eduardo Torroja, IETcc-CSIC, Serrano Galvache 4, Madrid 28033, Spain; E-Mails: mercesanc@ietcc.csic.es (M.S.M.); mcalonso@ietcc.csic.es (M.C.A.A.); 2 Programs Office, CSIC Delegation in Andalusia, Seville 41013, Spain; E-Mail: ana.hidalgo@csic.es; 3 NIR/MIR Spectroscopy Unit, Central Service for Research Support (SCAI), University of Cordoba, Campus de Rabanales, Cordoba 14071, Spain; E-Mail: nir@uco.es

**Keywords:** low-pH cements, hydration, microstructure, pH sensors, corrosion of steel reinforcements

## Abstract

Low-pH cements are designed to be used in underground repositories for high level waste. When they are based on Ordinary Portland Cements (OPC), high mineral admixture contents must be used which significantly modify their microstructure properties and performance. This paper evaluates the microstructure evolution of low-pH cement pastes based on OPC plus silica fume and/or fly ashes, using Mid-Infrared and Near-Infrared spectroscopy to detect cement pastes mainly composed of high polymerized C-A-S-H gels with low C/S ratios. In addition, the lower pore solution pH of these special cementitious materials have been monitored with embedded metallic sensors. Besides, as the use of reinforced concrete can be required in underground repositories, the influence of low-pH cementitious materials on steel reinforcement corrosion was analysed. Due to their lower pore solution pH and their different pore solution chemical composition a clear influence on steel reinforcement corrosion was detected.

## 1. Introduction

Low-pH cements have been developed to be used in underground repositories for high level waste. The research on low-pH cementitious materials was initially developed in Canada and Japan [1,2], and subsequently has been addressed from various approaches depending on the type of base cement used: (1) Calcium Silicate Cements (based on Ordinary Portland Cements); (2) Calcium Aluminate Cements (CAC based); (3) Phosphate Cements; and (4) Magnesia Cements [1,2,3,4,5,6,7,8,9,10]. If Ordinary Portland Cements (OPC) were to be used to produce concrete for underground repositories, their contact with the ground water would create pore water leachates with a pH > 13. The generation of this alkaline plume would have detrimental effects on the engineering bentonite barrier of the repository, as it has low stability at pH > 11 [11]. To limit this risk, low-pH cementitious materials are being developed to obtain a target pH < 11.

The development of low-pH cements based on OPC implies an important reduction of the OPC content used and its substitution for mineral admixtures with high silica content as silica fume (SF) or fly ashes (FA). These combinations follow the pozzolanic reaction that consumes portlandite [Ca(OH)_2_ or CH; the main phase responsible for the high pore solution pH] [6,7,8,10,12,13,14]. Therefore, the desired decrease in the pore solution pH is accompanied by significant changes in the hydrated phases, not only by a decrease in the portlandite content but also by forming different C–S–H gels which modify the standard properties of these materials [6,7,8,10]. So a deep understanding of the hydration processes of low-pH cementitious materials is needed. This paper deals with the microstructure of cement pastes based on binary and ternary binders with OPC plus SF and/or FA, and their evolution over time. Mid-Infrared and Near-Infrared spectroscopy have been used for this purpose.

Besides, once the lower pore solution pH values of these cement formulations were proven [10], embedded metallic sensors were used for *in situ* measuring and monitoring their pore water pH, in order to assess their real application in these special cements. Although several embedded pH sensors have been described in the literature, most of them have not been used in cementitious materials. The use of embedded sensors in cementitious materials requires that the sensors can withstand the harsh conditions of a high alkaline environment for a prolonged length of time [15].

As previously mentioned, a low-pH concrete should be compatible with the surrounding environment and hence its pH must be low enough to prevent the bentonite from being altered. However, if the disposal of high level wastes in underground facilities requires the use of concrete for structural support [16], thus implying the use of reinforced concrete, the steel reinforcement stability against corrosion could be compromised by these low pH values. It is well known that the high alkalinity of the pore solution in conventional OPC materials (pH > 12.6) promotes the formation of a stable passive layer of oxide that protects the rebar from a corrosion process. However, the stability of this passive layer can be affected by the decrease of the pore solution pH and the initiation of an active corrosion process can occur at pH values below 11, as occurs, e.g., in the case of concrete carbonation. Then, as the low-pH cement formulations strongly influence the pH value, the formation of this passive layer, as well as its long-term stability, can be affected. In this context, when the use of these formulations is considered for reinforced concrete, the durability of the reinforcements in these new materials with lower pH must be guaranteed [17]. Therefore, their susceptibility to corrosion should be analysed. In this paper, preliminary results are presented.

## 2. Experimental Section

### 2.1. Studies Made in Cement Pastes

Ordinary Portland Cement, silica fume and fly ashes were used for the testing program. Fly ash was classified as Class F according to the ASTM C618 definition. The chemical composition of the raw materials is presented in Table 1.

**Table 1:** Chemical composition of Ordinary Portland Cement (OPC) and mineral admixtures (% by weight).

Raw material	LI	IR	SiO_2_	Al_2_O_3_	Fe_2_O_3_	CaO (total)	MgO	SO_3_	Na_2_O	K_2_O	CaO (free)
OPC	4.19	1.09	17.4	4.68	5.08	60.3	1.78	3.17	0.18	0.34	1.85
SF	0.09	0.06	92.7	0.60	3.78	1.31	0.93	–	0.15	0.37	0.01
FA	2.19	0.52	54.3	26.9	5.38	4.52	2.24	–	0.63	3.17	0.15

LI: loss of ignition; IR: insoluble residue.

In the cement pastes formulation a deionized water to binder ratio (w/b) = 0.5 was used, and the samples were cured in a chamber at 98% RH and 21 ± 2 °C until the point of testing. Table 2 summarizes the formulations used in the different cement pastes, indicating the silica content of every combination and the pore solution pH measured at 90 d of curing using the Pore Fluid Expression Technique [18,19]. It is obvious that the higher the silica content in the binder, the lower the pore fluid pH measured.

**Table 2:** Cement formulations used in the pastes.

Sample	OPC (%)	SF (%)	FA (%)	SiO_2_ (%) total	pH (90 d)
Ref	100	–	–	18	12.9
B-1	60	40	–	47	12.2
B-2	50	50	–	55	11.2
T-1	35	35	30	51	11.2

The hydration evolution of the pastes was determined by stopping the hydration at different ages (2, 7, 30 and 90 d), by means of powdering the samples and removing the free water with ethanol and acetone (after powdering the cement paste ethanol is added to dry the sample and the sample is filtered under vacuum; after that, due to its lower vapour pressure, acetone is added to remove possible remaining ethanol and the sample is filtered again under vacuum thus totally drying the sample). Samples for Fourier Transform mid infrared spectroscopy (FTMIR) and Fourier Transform near infrared spectroscopy (FTNIR) analyses were dried at 60 °C over 24 h; then isolated until the tests were performed. Mid infrared spectra were taken at room temperature with a Bruker Tensor27 FTMIR spectrophotometer equipped with a DTGS detector and a CsI beam splitter. For each sample, 256 scans were recorded in the 4000–400 cm^−1^ spectral range with a resolution of 4 cm^−1^. Test samples were prepared by mixing powdered cement pastes with potassium bromide. Undried 12 mm diameter KBr pellets were used for measurements. Near infrared spectra were taken at room temperature with a Perkin Elmer Spectrum One FTNIR spectrophotometer (FT-NIR) equipped with a NIR DTGS detector and a CaF_2_ beam splitter. Sampling was performed in reflectance mode by means of an integrated sphere accessory using a Spectralon^©^ background (Labsphere, North Sutton, NH, USA). For each sample, 50 scans were recorded in the 10000–4000 cm^−1^ spectral range with a resolution of 8 cm^−1^. Approximately 1 g of test sample was poured into a glass petri dish base and the surface was pressed and smoothed with an aluminium slide. The spectral data were processed by means of the Opus (Bruker, Billerica, MA, USA) and Spectrum (Perkin Elmer, Waltham, MA, USA) software packages supplied with the instruments. For both, FTMIR and FTNIR, the background was measured before every analysis, in order to guarantee the spectra correction for environmental water.

The pastes were also observed by Backscattered Electron Microscopy with EDAX analyses for determining the C/S of C–S–H gels at 90 d of hydration. A JEOL JSM 5400 scanning electron microscope (SEM) equipped with a solid-state backscattered detector and a LINK-ISIS energy dispersive (EDAX) was used.

### 2.2. Studies Made in Mortars

Two types of studies were performed in mortars using two cement formulations: Ref and B-1. A binder: siliceous sand ratio of 1:3 was used. The w/b ratio was modified to obtain a similar fluidity (slump = 18 ± 1 cm) in both mortars (w/b = 0.5 in Ref sample and w/b = 0.65 in B-1 one; 2% wt binder of Naphthalene Formaldehyde base superplasticizer was added in both cases). Two cylindrical samples with two embedded steel reinforcements were fabricated for both types of mortars to confirm the repeatability of the tests. Steel reinforcements of 6 mm in diameter were used. The exposed area of the steel rebars was 5.65 cm^2^.

The samples were cured in a chamber at 98% RH and 21 ± 2 °C during the testing period. Electrochemical variables such as the corrosion potential (E_corr_) and the polarization resistance (R_P_) were monitored from the fabrication of the mortar samples until 300 d of testing in order to guarantee the long-term stability of the rebars. The corrosion current density (i_corr_) evolution was determined from R_P_ measurements as *i*_corr_ is inversely proportional to R_P_, *i.e.*, $i_{corr}=\frac{\text{B}}{{\text{R}}_{\text{P}}}$ , where *i*_corr_ is expressed as µA/cm^2^, R_P_ is expressed in kΩ·cm^2^ and B is the Tafel constant (a value of 26 mV is usually recommended for this constant). A three-electrode arrangement was used to carry out the R_P_ measurements: the steel rebar was the working electrode, a graphite rod of 5 mm in diameter and 6 mm in length was used as counter-electrode and the saturated calomel electrode was used as reference electrode. The measurement was carried out by a linear sweep with a scan of 10 mV/min between −20 mV to +20 mV of the corrosion potential. The ohmic drop was compensated in each measurement to avoid the influence of the mortar resistance in the R_P_ determination.

To match the obtained measurements with the pH values, the pore fluid pH of the fabricated mortars was also measured at different curing times following the method described in [20]. This method is based on *Ex Situ* Leaching (ESL) procedures whereby a powdered portion of the sample is mixed with an equivalent mass of deionised CO_2_-free water; the measurement of the pH is undertaken for the resulting suspension after stirring it vigorously and continuously for five minutes. Furthermore, two bismuth electrodes, sensible to pH changes in alkaline conditions [21], were embedded in each mortar sample in order to monitor the pH evolution. The bismuth electrode response was registered by measuring the open circuit potential *vs.* a saturated calomel electrode (SCE). A linear relationship with negative slope has been reported between pH and the bismuth electrode potential [22] with more anodic potential values for less alkaline conditions.

## 3. Results and Discussion

### 3.1. Evolution of the Microstructure of Low-pH Cement Pastes

Firstly, the results obtained by FTMIR are presented and, secondly, the FTNIR results. In both cases, due to the high mineral admixture contents of the low-pH cement pastes, the IR spectra of SF and FA are discussed to clarify the spectra obtained in the pastes.

#### 3.1.1. FTMIR Results

The MIR spectrum of SF (not shown) shows a band at 1120 cm^−1^ that corresponds to asymmetric stretching vibrations of Si–O–Si bridging sequences; another at 807 cm^−1^ corresponds to symmetric stretching vibrations of Si–O–Si bonds; and the last one at 477 cm^−1^ is associated with O–Si–O bond bending vibration [23]. The FA spectrum (not shown) has bands at 3450, 1085, 800, 553 and 465 cm^−1^. The band appearing at 553 cm^−1^ is associated with the octahedral aluminium present in mullite [24]. A band associated with symmetric stretching vibrations of Si-O-Si (from quartz) and Al–O–Si bonds (tetrahedral aluminum of mullite) appears at 800 cm^−1^ [23]. The bands around 1170–1130 cm^−1^ are associated with mullite vibrations and T–O vibrations in quartz. The band appearing at 460 cm^−1^ is associated with O–Si–O or O–Al–O bond bending vibrations.

**Figure 1:**
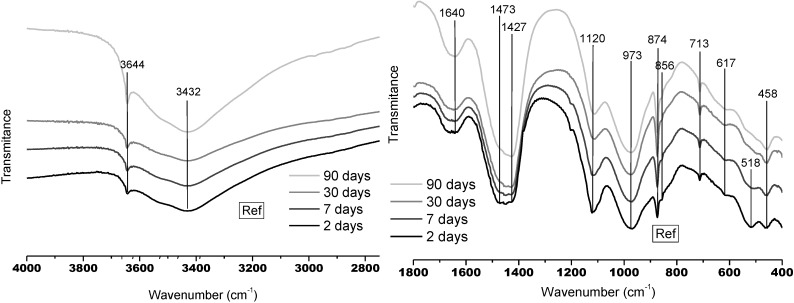
Fourier Transform mid infrared spectroscopy (FTMIR) spectra of Ref paste at 2, 7, 30 and 90 d.

Figure 1 shows the MIR spectra of the Ref paste at different hydration times. In this sample, portlandite and C–S–H gels are the main hydrates formed. In the OH-stretching region (~2800–4000 cm^−1^) there is a band at 3644 cm^−1^, corresponding to Ca–OH vibrations from portlandite, and a band at 3432 cm^−1^ that refers to stretching vibrations of O–H groups in H_2_O or hydroxyls with a wide range of hydrogen bond strengths [25]. It is evident that the “portlandite band” increases in intensity with the hydration time.

In the Ref paste, the main bands corresponding to C–S–H gels are detected at 973 cm^−1^ and 458 cm^−1^ [26], being the first ones assigned to Si–O stretching vibrations of the Q^2^ tetrahedra of C–S–H gels with jennite type structure that indicates high C/S (>1.2) ratios. The band at 610–620 cm^−1^ can be also associated with the presence of silicates or aluminosilicates gels as it is related to Si–O–Si or Al–O–Si symmetric stretching vibrations [27]. In the same way, the bands between 400 and 520 cm^−1^ are due to internal deformation of TO_4_ tetrahedra (T = Si or Al) [26]. Some characteristic SO_4_^2−^ bands from ettringite (calcium sulfoaluminate hydrate) are located at 1100 cm^−1^ and 610 cm^−1^ [28] but the first one can be obscured by the Si–O stretching band at 1120 cm^−1^ assigned to the presence of C–S–H gels [26]. Finally, many types of carbonated phases are also detected in the Ref paste but the disappearance of the 1473 cm^−1^ band and the increase in intensity detected with the hydration time in the bands at 1427 cm^−1^ and 713 cm^−1^, indicate that calcite is the main carbonated phase at longer hydration times [29,30].

**Figure 2:**
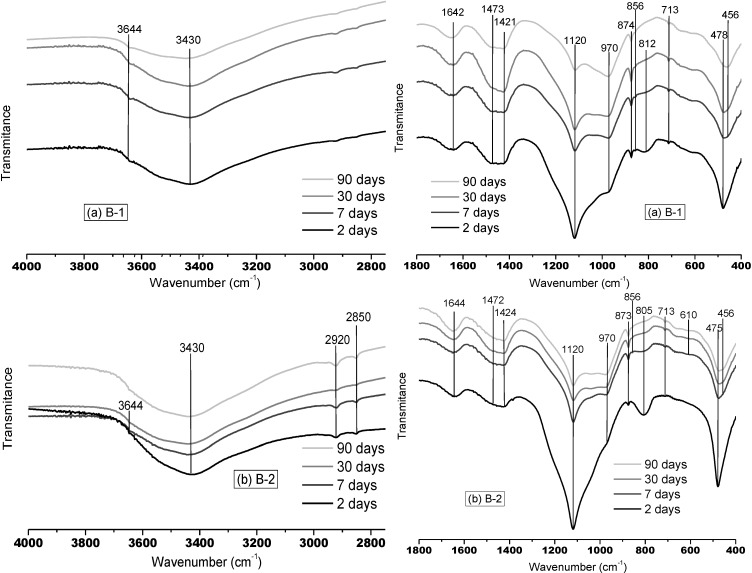

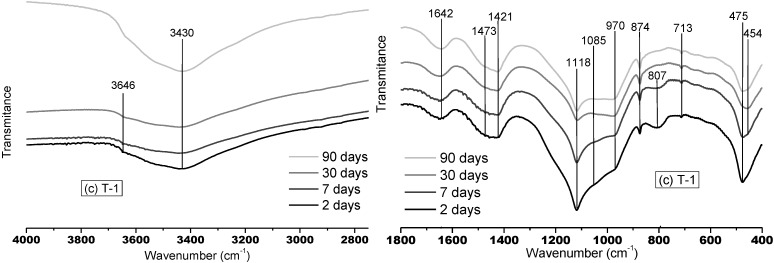
FTMIR spectra of binary pastes at 2, 7, 30 and 90 d. (**a**) B-1; (**b**) B-2; (**c**) T-1.

Many differences are detected in the MIR spectra of the low-pH cement pastes compared to the reference sample ones, mainly at longer curing times. Figure 2 shows the MIR spectra of the low-pH cement pastes at different hydration times.

In the OH-stretching region, disappearance of the “portlandite band” (3644 cm^−1^) with time is observed, and apparently its extinction. At 90 d, this band is only detected in the B-2 paste (the one with the less silica content; see Table 2), so the promotion of the portlandite consumption, due to the high mineral admixture contents used, is proven. In fact, the decrease in intensity of the 3644 cm^−1^ band with time is also followed by a decrease in the intensity of the peaks associated with the mineral admixtures (1120 cm^−1^, ~807 cm^−1^ and ~475 cm^−1^ in the pastes with SF, and also ~1085 cm^−1^ and ~460 cm^−1^ in the ternary binder with FA), indicating a consumption of these mineral admixtures due to the pozzolanic reaction.

Also in this region, higher amplitude of the 3450 cm^−1^ band attributed to hydrogen bonded OH species (O–H–O–H) is observed in the low-pH cement pastes. This fact can be related to the presence of C–S–H gels with a structure close to tobermorite. This structure implies low C/S ratios, higher polymerization of silicate chains and a high content of the less strongly hydrogen-bonded water molecules in the interlayer space [30]. The presence of C–S–H gels with tobermorite type structure in the low-pH cement pastes is also proven by observing the bands at 1120 cm^−1^ and 970 cm^−1^: the first one is generated by C–S–H gels with tobermorite type structure and the second one is directly associated to C–S–H gels with jennite type structure. It is evident that in the Ref paste (see Figure 1), the band at 970 cm^−1^ has a higher intensity than the one at 1120 cm^−1^ throughout the test period, suggesting a jennite type structure in the C–S–H phases formed. On the contrary, in the low-pH cement pastes the band between 1100 and 1200 cm^−1^ is more important, as the band at 970 cm^−1^ is merely anecdotal. However, the band at 1100–1200 cm^−1^ can also be overlapped by the 1120 cm^−1^ band related to SO_4_^2−^ from ettringite and/or by the main band of the SF (1120 cm^−1^) in the binary binders as well as by the main band of the FA (1085 cm^−1^) in the ternary paste.

However, there is other evidence of the presence of C–S–H gels with tobermorite structure in the low-pH cement pastes: the band around 450–460 cm^−1^, corresponding to internal deformation of TO_4_ tetrahedra, has a higher intensity in these pastes. It is true that quartz from SF or FA has a band at similar wavenumbers (470–460 cm^−1^), but it is narrower than the band around 450–460 cm^−1^ detected in the low-pH pastes spectra. The higher the intensity of this band, the higher the polymerization of the C–S–H gels, thus this band is mainly due to the existence of C–S–H gels, highly polymerized. This agrees with the formation of C–S–H phases with tobermorite type structure, as their polymerization is higher than that of the gels with jennite type structure [31]. Moreover, in the tobermorite type gels the possible substitution of Si by Al, generating an Al-tobermorite type structure and even forming C–A–S–H gels, cannot be rejected. In fact, this phenomenon was confirmed with the EDAX microanalyses presented at the end of Section 3.1.

Finally, as in the Ref sample, different carbonated phases were detected in the low-pH cement pastes, even at higher wavenumbers, between 2850 and 2950 cm^−1^ [25,29].

#### 3.1.2. FTNIR Results

The interpretation of the above absorption spectra in the mid-IR region (4000–400 cm^−1^) is complemented by the interpretation of reflectance spectra in the near-IR region (11,000–4000 cm^−1^) where overtones and combinations of fundamental stretching and deformation vibrations take place. The NIR spectra provide information on structural OH groups and H_2_O in cement hydrates. It must be said that the near infrared spectra of SF and FA (not shown) have a band between 4460 and 4500 cm^−1^ that can be assigned to the combination of stretching and bending vibrations of structurally bonded OH groups, and another band at 5100–5200 cm^−1^ typical of the combination of stretching and bending modes of H_2_O molecules.

**Figure 3:**
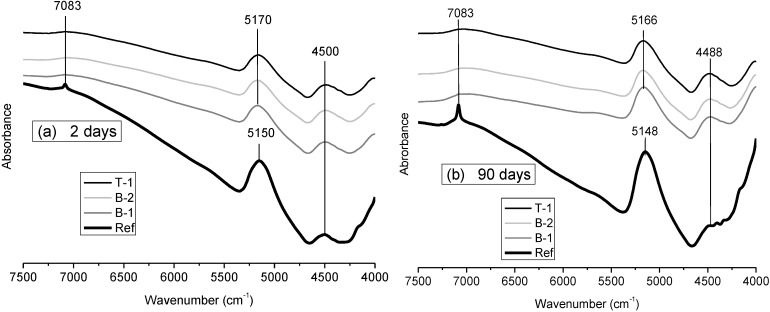
Fourier Transform near infrared spectroscopy (FTNIR spectra (4000–7500 cm^−1^) of the evaluated pastes at: (**a**) 2 d; (**b**) 90 d.

Figure 3 shows the FT-NIR spectra obtained at 2 d and 90 d of hydration in the cement paste combinations, considering the range between 4000 and 7500 cm^−1^, where the most characteristic bands of these type of cements appear. In this region three bands (or groups of bands) can be identified. The feature between 7000 and 7300 cm^−1^ is the first OH overtone (2ν_1_, 2ν_3_, and ν_1_ + ν_3_) and it has many components [32,33]: one easily detectable at 7083 cm^−1^ corresponding to OH stretching vibrations in portlandite, a broader but less precise shoulder at 7200 cm^−1^ related to C–S–H gel and a band around 7100 cm^−1^ related to the presence of Al-bonded O–H groups in ettringite. Although the overlapping of these bands/shoulders complicates the identification of the three components, the portlandite band is only recognizable in the Ref paste where increases its intensity from 2 to 90 d. However, in the low-pH cement pastes this band is not easily detectable and a very broad band related to structural water in C–S–H gels is observed, confirming the CH consumption.

The second important band is at 5150–5170 cm^−1^, arising from bending and stretching combinations of molecular water. Finally, there is a band around 4500 cm^−1^ that is the combination of the O–H stretching and the Si–OH stretching modes in the C–S–H gels which has higher intensity in the low-pH cement pastes throughout the test period. Yu *et al.* [26] analysed the FTNIR spectra of synthesized C–S–H gels with different C/S ratios, proving that the lower the intensity of the 4500 cm^−1^ band, the higher the C/S of the fabricated gels. Therefore, according to the results obtained in the present study it can be deduced that the low-pH cement pastes have C–S–H gels with C/S ratios lower than those measured in the Ref paste.

In fact, the EDAX microanalyses made on the evaluated pastes confirm the results obtained with FTMIR and FTNIR techniques. The C/S mean ratio measured in the Ref paste was 1.8 whereas those measured in the low-pH cement pastes were lower: 0.94 in B-1, 0.82 in B-2 and 0.75 in T-1. These C/S ratios agree with the recommendation made by Stronach and Glasser [34], who affirmed that to obtain a low pH in the cementitious materials the C/S ratio must be lower than 1.1. Besides, according to the pore solution pH values shown in Table 2, the lower the pore solution pH, the lower the C/S ratio of the C–S–H gel formed.

Moreover, comparing the EDAX microanalyses made in the C–S–H gels of the low-pH cement pastes with those made in the Ref paste, an increase in the alumina content is observed in the formers. This suggests the incorporation of Al in the C–S–H structure, even forming C–A–S–H gels, confirming the surmise made at the end of the previous section.

Therefore, whereas the Ref paste is mainly composed of portlandite and C–S–H gels with jennite type structure, the low-pH cement pastes are mainly composed of highly polymerized C–S–H or/and C–A–S–H gels with tobermorite type structure, and the portlandite is totally consumed at 90 d of curing (or even before). These different microstructures could present new interesting properties in low-pH cementitious materials for the possible retention of alkalis or other ionic species, topics assessed by different authors [8,10,35,36]. In fact, Hong and Glasser [35] demonstrated that the alkali binding process is more effective when the C/S ratios of C–S–H gels are between 1.2 and 0.85.

### 3.2. *In-Situ* Monitoring of the Pore Solution pH Evolution of Low-pH Mortars Using Embedded Metallic Sensors

**Figure 4:**
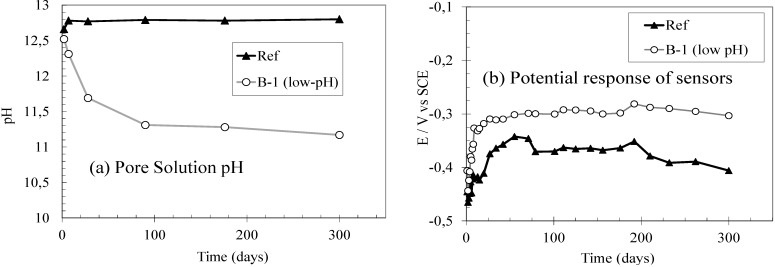
(**a**) Pore fluid pH evolution of the fabricated mortars; (**b**) Potential response of embedded metallic sensors.

The microstructural changes observed in the low-pH cement pastes are followed by a decrease in their pore solution pH to values near 11, the target value that allows the use of this type of materials in underground repositories of high level waste. Due to this main prospective use of these special cements (underground facilities), the feasibility of monitoring their pore fluid pH by using embedded metallic sensors has been preliminarily analysed in the present study. Figure 4a shows the pore fluid pH evolution of the fabricated mortars (measured using the method described in [20]) and Figure 4b, for comparison, the measurements obtained with the embedded sensors, both up to 300 d.

When observing the pore solution pH evolution of both types of mortars it is clear that the low-pH cement formulation used in this study (B-1) strongly reduces the pore solution pH during the first 90 d, maintaining this stable value long term. This same pH evolution was also observed with the embedded metallic sensors as shown in Figure 4b. The different stable pH values measured for both mortar mixes were expressed as different stable values of potential with the embedded sensors; as expected, more anodic potentials were registered for the lower pore fluid pH. Besides, a clear relation between the electrode potential and the pH value can be deduced, obtaining a mean potential value of −0.404 V (±0.021 V) for pore solution pH values above 12.6 (those measured in the Ref mortar) and potential values around −0.256 V (±0.002 V) for pore solution pH values around 11.3 (those measured in the low-pH mortar after 90 d). Nevertheless, an increase of the solution resistance is expected to be associated to the pore fluid pH decrease and further studies should be considered in order to analyse the influence of this change on the solution resistance.

### 3.3. Influence of the Use of Low-pH Cements in the Corrosion Performance of Steel Reinforcements

**Figure 5:**
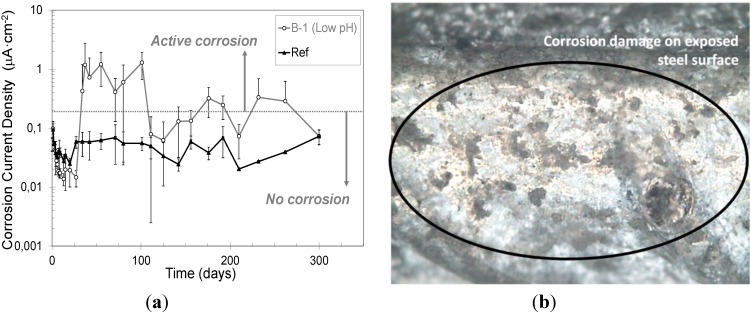
(**a**) Corrosion current density evolution; (**b**) Optical microscopy of corroded steel surface (×3).

As defined in the introduction, the disposal of high level wastes in underground repositories often requires the use of reinforced concrete for structural support. Therefore, when the reinforcements are embedded in low-pH cementitious materials, the formation of the protective passive layer on the reinforcement surface as well as its susceptibility to corrosion must be analysed. Figure 5a shows the corrosion current density evolution (*i*_corr_) for steel reinforcements embedded in both mortar mixes. The mean value of four reinforcements is represented, as similar evolution was observed in each mortar type (the standard deviations of the measured values are also shown as error bars).

Initially, low values of corrosion current density with decreasing tendency were registered, indicating the formation of a passive layer in both mortar mixes. However, in the case of the low pH mortar, a significant increase of i_corr_ was detected after 25 d of exposure, when the pore fluid pH was lower than 11.8 (see Figure 4a). After this time, *i*_corr_ values above the limit considered for passivation (0.2 µA/cm^2^) [37] are maintained. This fact is associated with the initiation of an active corrosion process with periods of passivity and reactivation, probably because the pH is at the limit between the active/passive states. In Figure 5b a magnified photography of the corroded steel surface is included as an example of the corrosion action. In this picture small corroded areas, extending along the whole steel surface, can be observed.

According to these results, alternative reinforcements or some additional protection measures against corrosion must be considered when low-pH cements are used in reinforced concretes for high level waste repositories where long-term life is mandatory.

## 4. Conclusions

From the results of this work several conclusions can be highlighted:

1. Mid- and near-infrared spectroscopy techniques were used in the study of the hydration of low-pH cement pastes based on OPC plus mineral admixtures, enabling the identification of their main solid phases. Whereas the Ref paste is mainly composed of portlandite and C–S–H gels with a jennite type structure and high C/S ratios, the low-pH cement pastes are mainly composed of high polymerized C–S–H or C–A–S–H gels with a tobermorite type structure (with C/S ratios below 0.95 according to the EDAX microanalyses made). In the low pH cement pastes the portlandite is totally consumed at 90 d of curing, or even before.
2. The feasibility of monitoring the pore solution pH of low-pH mortars by using embedded metallic sensors was preliminary proven. The different stable pH values measured for conventional or low-pH mortars are expressed as different potential stable values with the embedded sensors: more anodic potentials are registered for the lower pore fluid pH.
3. The susceptibility to corrosion when the steel reinforcements are embedded in low-pH cementitious materials was also analysed. In low-pH mortars a significant increase of i_corr_ values is detected after 25 d of exposure, when the pore fluid pH is lower than 11.8. After this time, i_corr_ values above the limit considered for passivation (0.2 µA/cm^2^) are maintained, as this fact is associated to the initiation of an active corrosion process.
